# Recent Advance in Biosensors for microRNAs Detection in Cancer

**DOI:** 10.3390/cancers3021877

**Published:** 2011-04-08

**Authors:** Silvia Catuogno, Carla L. Esposito, Cristina Quintavalle, Laura Cerchia, Gerolama Condorelli, Vittorio de Franciscis

**Affiliations:** 1 Istituto per l'Endocrinologia e l'Oncologia Sperimentale del CNR “G. Salvatore”, Via S. Pansini 5, 80131 Naples, Italy; E-Mails: silviacatuogno@libero.it (S.C.); c.esposito@ieos.cnr.it (C.L.E.); cerchia@unina.it (L.C.); 2 Dipartimento di Biologia e Patologia Cellulare e Molecolare, University of Naples “Federico II”, Naples, Italy; E-Mail: cristina.quintavalle@unina.it; 3 Facolta di Scienze Biotecnologiche, University of Naples “Federico II”, Naples, Italy; E-Mail: gecondor@unina.it

**Keywords:** microRNA, biomarker, cancer

## Abstract

MicroRNAs (miRNAs) are short non-protein-coding RNA molecules that regulate the expression of a wide variety of genes. They act by sequence-specific base pairing in the 3′ untranslated region (3′UTR) of the target mRNA leading to mRNA degradation or translation inhibition. Recent studies have implicated miRNAs in a wide range of biological processes and diseases including development, metabolism and cancer, and revealed that expression levels of individual miRNAs may serve as reliable molecular biomarkers for cancer diagnosis and prognosis. Therefore, a major challenge is to develop innovative tools able to couple high sensitivity and specificity for rapid detection of miRNAs in a given cell or tissue. In this review, we focus on the latest innovative approaches proposed for miRNA profiling in cancer and discuss their advantages and disadvantages.

## 1. Introduction

**Figure 1.**
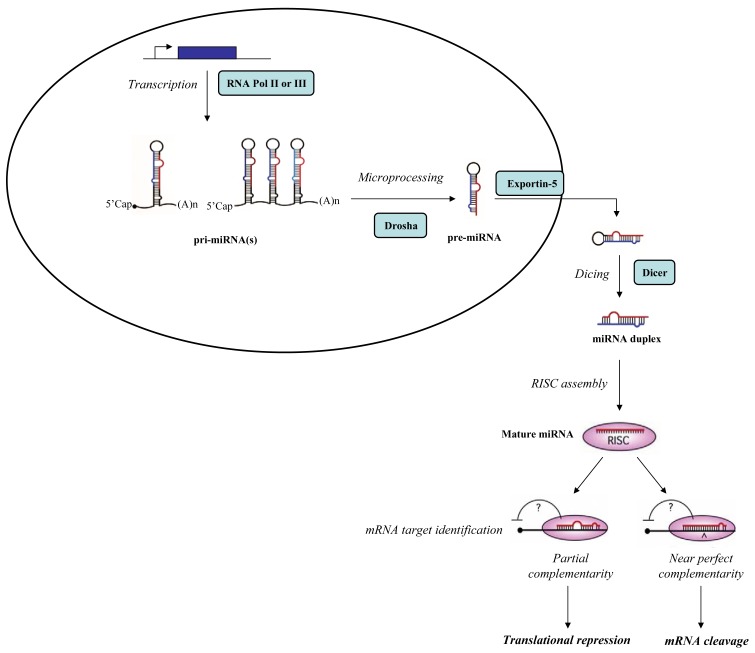
Scheme of miRNA processing pathway.

MiRNAs are a class of small (19–25 nucleotides) non-coding RNA molecules widely conserved through evolution. MiRNAs are first transcribed into long primary miRNAs (pri-miRNAs) by polymerase II or, in few rare cases, by polymerase III. Typically, pri-miRNAs display a 33 bp stem and a terminal loop structure with flanking segments. Primary miRNAs processing begins in the nucleus where an RNAse III enzyme, Drosha, in complex with other proteins processes the pri-miRNAs, inducing the conversion into precursor miRNAs (pre-miRNAs). Pre-miRNAs are 60–70 nt long hairpin RNAs with 2-nt overhangs at the 3′ end. They are transported into the cytoplasm by exportin-5, a RanGTP-dependent dsRNA-binding protein, and subsequently processed by Dicer, a cytoplasmic endonuclease RNAse III enzyme, that generates a miRNA duplex. The functional strand of the mature miRNA is then incorporated into the RISC (RNA-induced silencing complex), a ribonucleoprotein effector containing a catalytic endonuclease core, Argonaute2 (Ago2); Dicer; a dsRNA-binding protein named transactivating response RNA-binding protein (TRBP) and a protein activator of protein kinase R (PACT). The RISC mediates the degradation or the translation inhibition of the target mRNA (Figure 1). To produce the functional mature miRNA the duplex generated after Dicer-mediated cleavage need to be unwound, but to date it remains unclear when (*i.e.*, before or after RISC loading) and how the two strands are separated [1,2].

Recent studies have shown that miRNAs are involved in different biological processes and pathological states, particularly in the development of several cancers. Altered expression levels of miRNAs have been correlated with cancer type, tumor stage and response to treatments [3]. Thus, miRNAs represent a new class of promising diagnostic and prognostic biomarkers as well as new targets for cancer therapy [4,5].

On this basis, it is important to develop analytical methods for rapid and sensitive identification of miRNAs present in a particular cell or tissue or fluids (such as serum and plasma) samples. A key issue is the ability to distinguish between the precursor and mature form of a miRNA, since the mature form is the functional one and cellular levels of miRNA precursors does not necessarily correspond to cellular concentration of functional miRNAs [4].

To date, several methodologies have been applied to profile miRNAs including Northern blotting, *in-situ* hybridization, oligonucleotide microarrays, quantitative Reverse-Transcription-Polymerase Chain Reaction (qRT-PCR) and deep-sequencing [6]. Although Northen blot and *in-situ* hybridization continues to be used as the standard methods, these detection approaches have low sensitivity and generally require many steps, resulting laborious time-consuming procedures that are difficult for routine miRNA analysis.

In this review, we analyze in detail the recent advances in microarray-based detection platforms and next generation methods based on nanotechnologies. Innovative qRT-PCR, amplification and enzymatic-based methods as well as deep sequencing strategies are also discussed. In addition, in the final section we focus on the detection and characterization of circulating miRNAs as cancer biomarkers.

## 2. Alterations of miRNAs in Cancer

MiRNAs are expressed in a tissue-specific manner and changes in miRNA expression within a tissue type can be correlated with disease status. The tissue concentrations of specific miRNAs have been associated with response to therapy, metastatic potential and other clinical features in various types of cancer [5,7].

The first evidence for miRNAs involvement in human cancer comes from a study by Calin *et al.* [8], examining a recurring deletion at chromosome 13q14 to search for a tumor suppressor gene involved in chronic lymphocytic leukemia (CLL). This study describes that the region of deletion encodes two miRNAs, miR-15a and miR-16-1. Subsequent investigations have confirmed the involvement of these two miRNAs in the pathogenesis of CLL [9]. Furthermore, Constinean *et al.* reported that a miRNA by itself can induce a neoplastic disease [10]. In fact, by using a transgenic mouse model, they demonstrated that overexpression of miR-155 in B cells induce lymphoma pre-B leukemia.

Several other miRNAs dysregulated in different human cancer types have been reported. For example, it has been demonstrated that let-7 family contains miRNAs regulating the RAS family of oncogenes [11]. Petrocca *et al.* [12] showed that the miR-106b-25 cluster plays a key role in gastric cancer interfering with proteins involved both in cell cycle and apoptosis. In other studies, miR-155 was found overexpressed in Hodgkin lymphoma, pediatric Burkitt lymphoma and diffuse large B-cell Lymphoma [13-15]; miR-143 and miR-145 were significantly downregulated in colon cancer tissue compared with colonic mucosa [16]; miR-21 was overexpressed in many tumors [7], including glioblastoma [17], cholangiocarcinoma [18], multiple myeloma cells [19] and breast cancer [20,21].

Moreover, studies that investigated the expression of the entire microRNAome in various human solid tumors and hematologic malignancies have revealed differences in miRNA expression profiling between neoplastic and normal tissues [9,22-24]. These studies indicate that neoplastic tissues may be distinguished by the expression of specific signatures of as few as 20–30 different miRNAs and expression profiles may be highly predictive for the degree of response to several therapeutic agents [25]. Further, being much shorter than mRNAs, miRNAs are less vulnerable to degradation by ribonucleases and, unlike proteins, are not post-synthetic structural modified, and therefore easier to detect.

MiRNAs play a key role also in tumor metastasis. Indeed, for example miR-139 suppresses metastasis of hepatocellular carcinoma [26], while miR10-b was found highly expressed in metastatic breast cancer cells [27] even if its clinical utility is still questioned [28].

More recently, many evidences are emerging that tumor-derived miRNAs are present and detectable in serum, plasma, urine and other human body fluids (Table 1). Because of their abundance, tissue specificity and relative stability, circulating miRNAs hold a great promise as noninvasive or minimally invasive biomarkers in cancer [29,30].

## 3. Methods for miRNA Detection

Various strategies for miRNA detection have been developed. Here we discuss some of the most innovative ones remarking their advantages and limits (Table 2). Moreover we analyze methods used for detection and characterization of circulating miRNAs as new highly promising biomarkers for cancer diagnosis.

### 3.1. Microarray-Based Methods

To date the most widely used techniques in literature to study the expression profile of miRNAs in cancer, are based on microarray analysis. These approaches are particularly attractive for miRNA profiling since they allow multiplexed detection of miRNAs [40].

Microarray technologies are based on the hybridization between target molecules and their respective complementary probes. Oligonucleotide probes are immobilized on a support platform through a covalent link and fluorescent labeled miRNAs are hybridized with the array. The specific link between miRNAs and probes generates fluorescent signals that are revealed and quantified as discrete spots on the slide. This technique is very attractive because it allows the analysis of a large number of miRNAs at the same time obtaining a miRNA expression profile of specific cancer samples.

The trickiest steps in microarray analysis are the design of probes used for capture of miRNA molecules and labeling procedure of biological samples. Several modifications in both these steps have been introduced during the last years that have permitted to improve this technique.

The probe design is influenced by a number of matters related to the nature of miRNAs. Indeed, miRNAs are small molecules that represent only a tiny fraction of total cellular RNA with many of them belonging to the same family and differ only by few nucleotides. These characteristics make it difficult to design multiple probes with a suitable melting temperature (Tm), thus optimizing hybridization conditions without compromising specificity. Moreover, because there are often hundreds to thousands of probes in the same miRNA microarray, Tm normalization is absolutely required.

Different strategies have been proposed to overcome these problems. Recently locked nucleic acids (LNA) [41,42] have been used to increase melting temperature, probe affinity for its target and mismatch discrimination. This approach provides high sensitivity and specificity. Otherwise, Baskerville *et al.* reported a strategy for Tm normalization by adjusting the length of the probes. In this method, appropriate adaptor sequences are linked to either one or both ends of the miRNA molecules and, based on the adaptor sequence, the probe is suitably lengthened or appropriately truncated if the original Tm is either too low or too high [43].

The procedure used for miRNAs labeling is another pivotal step for the success of microarray analysis. Different ways for direct or indirect labeling of miRNAs have been proposed [44]. Indirect methods are based on the labeling of the reverse transcribed miRNA or the RT-PCR product. This increases the labeling stability and sensibility. Direct methods (such as the use of guanine reagents, T4-RNA ligase or chemical labeling) are usually easier to use and help to avoid errors introduced by the reactions of reverse transcription and PCR amplification, even though they require a considerable amount of RNA (in the order of micrograms).

To date, various companies (such as Affymetrix, Inc., Santa Clara CA, USA; Agilent Technologies, Inc., Santa Clara CA, USA; Applied Biosystems, Inc., Foster City CA, USA; Exiqon A/S, Vedbaek Denmark; and Rosetta Genomics, Inc., Rehovot, Israel) provide different microarray platforms for miRNA detection with a great potential applicability in clinical field.

### 3.2. Nanotechnology-Based Methods

In recent years different strategies based on nanoparticles have been developed for mature miRNA profiling. All these methods are direct approaches able to minimize artifacts due to sample amplification and labeling.

A number of nanotechnology-based approaches proposed in recent years are based on the use of electrical detection techniques. In a first approach Gao Z. and Yang Z. [45] employed the Elettrocatalytic Nanoparticle Tags (ENT) strategy that is based on amplified chemical ligation utilizing an indium tin oxide electrode and isoniazid-capped OsO_2_ nanoparticle tags. MiRNAs are oxidized with sodium periodate and then hybridized to DNA capture probes on the electrode. The signal is then chemically amplified through a ligation reaction to tag miRNAs with the OsO_2_ nanoparticles that efficiently catalyze the oxidation of hydrazine resulting in an electrocatalytic activity at 0.10 V. This procedure greatly enhances the sensitivity leading to detect amounts of miRNAs as low as 80 fM. The assay was successfully applied to analyze let-7b, mir-106 and mir-139 in total RNA extracted from HeLa cells reducing the amount of sample needed to nanograms [45]. The same group developed another electrical detection strategy based on a microscopic platform made with interlocking gold and titanium microelectrodes with wells in between. Capture probes of peptide nucleic acid (PNA) are chemically fixed into these wells and hybridized with the target miRNAs. The anionic nature of the miRNA phosphate backbone then catalyzes the formation of polyaniline (PAn) nanowires from a solution of cationic aniline particles, so that the conductance of the deposited PAn nanowires correlates directly with the amount of the captured miRNAs. By using total RNA extracted from different cancer cell lines, the target miRNAs can be quantified in a range from 10 fM to 20 pM with a detection limit of 5.0 fM [46].

More recently Peng *et al.* [47] further ameliorated this strategy proposing a novel protocol enabling electrical detections with minimal background. In this approach, target miRNAs are tagged with RuO2 nanoparticles that serve as a catalyst for the polymerization of aniline, allowing selective PAn deposition exclusively at the hybridized miRNA strands thus producing a clean background and a high signal-to-noise ratio.

An innovative nanotechnology-based method that utilizes Surface Plasmon Resonance Imaging (SPRI) has been developed by Fang *et al.* [48]. They described a novel approach that combines surface poly(A) enzyme chemistry and nanoparticle-amplified SPRI measurements for the miRNA detection on LNA microarrays. The target miRNAs are hybridized on LNA microarray and poly(A) tails are added to the miRNAs by poly(A) polymerase reaction. Poli(T) oligo-modified gold nanoparticles (GNPs) are then adsorbed onto poly(A) tails for signal amplification and subsequently detected with SPRI. This methodology can be used to measure miRNAs present in total RNA samples with excellent sensitivity at attomole levels, resulting about 50 times more sensitive than the fluorescence-based microarray [48].

In order to develop a simple read-out and high sensitive method that does not require expensive equipment, Yang *et al.* [49] proposed a colorimetric approach based on gold nanoparticles. This method utilizes two probes, a biotinylated probe (capture probe) and a gold nanoparticle probe hybridized to the complementary target miRNAs in a sandwich assay format. The complex is then immobilized onto the surface of a streptavidin-coated microplate and the signal of absorbed gold nanoparticles is amplified by silver enhancement and recorded with colorimetric absorbance by a microplate reader. By this method, distribution of miR-122a/miR-128 in total RNA from mouse brain and liver tissue was detected and synthetic miRNA-122a was quantified with a detection limit of 10 fM miRNA or 2 ng of total RNA [49].

Recently, the Raman enhancing property of GNPs has been exploited to develop a surface-enhanced Raman scattering (SERS)-based assay for miRNAs. However, SERS platforms based on GNPs has proven to be not useful for quantitative diagnostic assays due to low reproducibility [50]. To overcome this problem, alternative SERS enhancing substrates have been proposed. Driskell J. D. *et al.* examined a silver nanorod (AgNR) array prepared via oblique angle vapor deposition [51-53]. In their approach miRNA sequences are incubated with the silver nanorod array SERS substrate and SERS spectrum was analyzed. Different synthetic miRNAs (such as let-7 miRNAs, miR-16, miR-21, miR-24a, miR-133, miR-218 and miR-224) were analyzed with high specificity [53].

To date the number of new strategies that employ nanomaterials are growing rapidly [54,55], revealing these approaches to be the most promising for the development of miRNAs-based prognostic and diagnostic tools in cancer.

### 3.3. qRT- PCR-Based Methods

Among the several advantages of qRT-PCR, widely used for gene expression quantization [56,57], are the high level of sensitivity (only few picograms of starting material are needed), accuracy and practical ease that make qRT-PCR a powerful tool for miRNA detection as well.

On the other hand, the main limit to extend this method to miRNA detection is represented by the very short length of mature miRNAs. In fact, the first approach used allowed to detect and quantize precursor molecules rather than mature miRNAs [58].

A stem-loop qRT-PCR based on TaqMan assay was developed by Chen and colleagues from Applied Biosystems and is currently commercialized [59]. This approach, obviously, shows all the advantages of conventional TaqMan qRT-PCR, such as sensitivity (only 25 picograms of starting RNA are needed), but it involves the use of a stem-loop primer during the reverse transcription reaction. Such an approach is specific for mature miRNA identification and allows discriminating between strictly related miRNAs. This method is also better than conventional TaqMan qRT-PCR in terms of reverse transcription efficiency and specificity. To date, stem-loop qRT-PCR is successfully and widely utilized to detect miRNA dysregulation in different cancer types [60-63].

In the same year, Raymond *et al.* [4]developed a very sensitive (femtomolar concentrations of starting RNA) SYBR Green qRT-PCR for the detection of mature miRNAs using Locked Nucleic Acid (LNA)-modified primers. Both stem-loop and SYBR Green qRT-PCR methods have the disadvantage to be quite costly.

To develop a new cost-effective qRT-PCR approach for mature miRNA detection, Sharbati-Tehrani *et al.* [64] proposed a highly specific and sensitive method (called miR-Q), which neither requires the use of fluorophore probes, nor LNA-modified oligonucleotides. MiRNAs are first reverse transcribed and simultaneously elongated using a miRNA-specific oligonucleotide with 5′ overhang and then cDNA molecules are amplified using three DNA-oligonucleotides at different concentrations. This approach has been utilized to quantify miRNAs in different cancer cell lines and then for miRNA expression profiling of *in vitro*-fertilized bovine embryos [64,65]. A very simple and convenient method is based on Poly(A)-Tailed Universal Reverse Transcription [66,67]. In this approach total RNA is first polyadenylated by poly(A) polymerase and then cDNA is synthesized by using a specific primer containing oligo dTs flanked by an adaptor sequence. Finally, the cDNA is amplified using a miRNA-specific primer and a universal primer.

The above approaches are all low throughput methods. However in more recent years these approaches have been modified by high-throughput miRNA profiling. For example, Applied Biosystems Inc. provide TaqMan Low Density Array cards that simultaneously quantifies hundreds of miRNAs by TaqMan qRT-PCR reactions using Megaplex™ stem-loop primer pools for the miRNA reverse transcription step. Furthermore, Signosis Inc. has developed a highly sensitive and specific platform that combine oligo-ligation and SYBR green based qRT-PCR for multiple miRNA detection. These platforms are particularly attractive since they can be used extensively for clinical diagnosis.

An example to simultaneously quantify different miRNAs in the same qRT-PCR reaction is the use of innovative probes, named molecular beacons. Molecular beacons are single-stranded probes with a stem-loop structure that recognize a specific target molecule [68,69]. The complementary sequence to the target is in the loop of the molecule, while the stem is formed by the annealing of two complementary sequences with a fluorophore linked to the end of one arm and a quencher linked to the end of the other one. Molecular beacons emit fluorescence only when they hybridize with the target, undergoing a spontaneous conformational reorganization that forces the fluorophore and the quencher to move away from each other. This approach is very sensitive to mismatches and, since probes can be linked with different fluorophores, is also helpful to simultaneously detect different target miRNAs. Molecular beacons have also been modified [70] to specifically quantify mature miRNAs. In this case when the probes hybridize with pre-miRNA or pri-miRNA, its fluorescence is quenced by a guanine in the target sequence, while hybridization of the probe with mature miRNA which has no complementary guanine results in fluorescent emission. This approach has been recently utilized for detection of miRNAs overexpressed during myogenic differentiation [71].

### 3.4. Amplification-Based Methods

Here we discuss some promising miRNA detection strategies that require a PCR amplification step but do not involve real time quantitative analysis.

The padlock-probes and rolling-circle amplification technology was initially developed by Nilsson *et al.* [72,73] and more recently improved by Jonstrup *et al.* [74] for detecting and quantifying miRNAs. Padlock probes are linear DNA probes where the terminal sequences are designed to be exactly antisense to the 5′-end and the 3′-end of a specific miRNA. After annealing with the miRNA the padlock-probe is circularized by a DNA ligase and then the miRNA is used as primer for rolling circle amplification. The method is very sensitive to mismatches and has the power to discriminate between closely related miRNAs. Moreover this technology is highly quantitative, specific and very inexpensive, since no specific equipments required. This approach has been successfully used to quantify different miRNAs including miR-16, miR-17-5p, miR-20a, miR-21, miR-27a and miR-92 [74].

Another example of amplification-based approach is the bead-based flow cytometric miRNA expression profiling [75]. In this approach miRNAs are first processed with a ligation reaction that adds adapter oligos to both 3′ and 5′ ends and then reverse transcribed with primers complementary to the adaptor oligos. The resulting cDNA is amplified with biotinlyated forward primers, PCR products are hybridized with specific probes on fluorescent beads and finally analyzed by flow cytometry. This method has high accuracy, low costs and is useful for high-throughput miRNA profiling. This technique has been successfully applied to carry out a systematic expression analysis of 217 mammalian miRNAs from 334 samples, including multiple human cancers [75]. In this work the data from the bead-based miRNA profiling allowed to distinguish tumors of different developmental origin and at different stages. Thus bead-based flow cytometric miRNA expression profiling seems to be a very interesting diagnostic tool for cancer, since it is also easy to implement in a routine clinical setting.

### 3.5. Enzymatic-Based Methods

In this section we analyze examples of miRNA profiling strategies that involve enzymatic activities different from DNA polymerase.

A first promising approach included in this category is the splinted ligation method. It was originally developed by Moore and Query [76] and then Maroney *et al.* [77,78] adapted the technology to detect and quantify miRNA expression. Splinted ligation method is a sensitive and simple approach that also allows simultaneous processing of multiple samples. This method is based on the use of a couple of oligonucleotides named, the *bridge* and the *ligation* oligonucleotide respectively. The bridge oligonucleotide hybridize with the target miRNA and with a 5′-end-radiolabeled ligation oligonucleotide. This allows the formation of a double-stranded structure with a nick on one strand that is ligated by T4 DNA ligase, thus labeling the template miRNAs while the unligated oligonucleotides is treated with a phosphatase to remove the 5′-end labeling. Following the splinted-ligation reaction, labeled miRNAs and any residual-labeled ligation oligonucleotides can be separated and analyzed by denaturing gel electrophoresis. This approach has been successfully validated for different miRNAs including miR-21, miR-1, miR-9, miR-16, miR-20a, miR-21, miR-26a, miR-124a and miR-37 [77,78]. The main disadvantages of this technique are the use of radioactive labeling and the relatively low sensitivity. Other enzymatic-based methods that avoid the use of radioactive labeling have been proposed.

For example, the enzyme-linked oligonucleotide assay (ELONA) was developed by Mora and Getta [79]. This assay is based on the use of miRNA probes and miRNAs labeled at the 3′ end with a 31-base oligonucleotide complementary to the outer arms of horseradish peroxidise (HRP)-conjugated DNA dendrimers. MiRNA probes are first spotted onto microtiter plates and then are hybridized with labeled miRNAs. HRP-conjugated dendrimers are finally used as detection molecules for signal amplification. ELONA takes very short experimental time, it is no expensive and it has a high sample throughput, but it has relatively low sensitivity (require more than 30 ng/well of sample). This approach has been successfully used to quantify tissue-specific expression of miR-1 in heart tissue, miR-122 in liver and miR-124a in brain [79].

An alternative to ELONA is the Bioluminescence miRNA detection method [80]. It is a competitive solid-phase hybridization-based method that makes use of the bioluminescent protein *Renilla* (Rluc) as label. This method is simple, rapid and sensitive with a detection limit in the order of 1 fmol. This approach has been successfully applied for determination of miR-21 in both human breast adenocarcinoma MCF-7 and non tumorigenic epithelial MCF-10A cellular extracts [80].

Other interesting enzymatic-based methods are the RNA-primed Array-based Klenow Enzyme assay (RAKE) [81] and the invader assay [82]. RAKE is a new method for high-throughput miRNA detection that is even more specific than other microarray-based expression profiling platforms. An oligonucleotide with a 5′ spacer, covalently linked onto a glass platform, followed by a antisense probe complementary to the target miRNA that permits forming double stranded hybrids. Following exonuclease I degradation of unbound single stranded oligos, the miRNA bound to the probe is used as primer for Klenow fragment of DNA polymerase I, which catalyzes the addition of biotin-conjugated dATP onto the spacer template with an increase of the signal without any need of PCR amplification of the template. RAKE assay was used to profile miRNAs from normal human adult and fetal brains and from reactive astrocytosis and oligodendroglial tumors. It is also a sensitive method since it requires as low as 10 pg of starting RNA [81].

The invader assay is a detection technology developed by Allawi *et al.* [82] and based on the use of a structure-specific 5′ nuclease (Cleavase) and a final fluorescence measurement. The miRNA target is first hybridized with a specific probe and an Invader oligonucleotide, designed to form an overlap-flap structure, substrate of the Cleavase. After the cleavage the flap fragment is released and a second overlap-flap structure is formed by the annealing of the flap fragment and a FRET oligonucleotide, linked to a fluorophore and a quencher, to a secondary reaction template. Then the Cleavase separate the fluorophore from the quencher on the FRET oligonucleotide, thus generating a fluorescence signal. This is a very simple and rapid approach (requiring only 2–3 hours of incubation), able to discriminate between precursor and mature miRNAs and also between closely related miRNAs, but it has the disadvantage to be less specific and sensitive than qRT-PCR-based methods (at least 50 ng of total RNA as template are required). Invader assay has been successfully applied for the detection of several miRNAs in cancer [13] and has been also modified for high-throughput miRNA profiling [83,84].

### 3.6. Deep Sequencing-Based Methods

In recent years innovative deep sequencing technologies, originally used for genomic sequencing [85], have been also applied for simultaneous sequencing of up to millions of miRNA molecules. These include the 454 Genome Sequencer (Roche Applied Science, Basel CH) based on pyrosequencing, the Illumina Genome Analyzer (Illumina Inc., San Diego, CA, USA) based on Solexa technology and the SOLiD platform (Applied Biosystems, Inc., Foster City, CA, USA), a ligation-based sequencing. For all different high-throughput systems small RNA cDNA library preparation is a critical point and the following basic steps are required: (1) total RNA isolation; (2) small RNAs enrichment; (3) 3′ and a 5′ adaptor ligation (platform-specific); (4) reverse transcription; (5) PCR amplification by minimal rounds to avoid library bias; (6) sequencing [86].

In the 454 and in the SOLiD technologies an adaptor-flanked library is amplified by an emulsion multi-template PCR using a single primer pair, corresponding to the adaptor sequences. One PCR primer is 5′-linked to the surface of micron-scale beads, included in the reaction. After PCR amplification, each bead will bear on its surface PCR products corresponding to a single molecule from the template library. These clonally amplified beads can then be used as template for features for 454 or SOLiD sequencing platform.

With the 454 platform beads are randomly deposited on the wells of a microarray and sequenced by pyrosequencing. In this approach in each cycle a single nucleotide is introduced and then a substrate (luciferin, adenosine 5′-phosphosulphate) is added to produce light signal at wells where polymerase drives the incorporation of the nucleotide.

Instead, with the SOLiD platform, beads are used to create a disordered, dense array of sequences and in each sequencing cycle is introduced a partially degenerate population of fluorescently labeled octamers. In this population the label correlates with the identity of the central 2 bp of the octamer. Several such cycles will iteratively interrogate an evenly spaced, discontiguous set of bases.

Finally in the Solexa technology an adaptor-flanked library is amplified by a bridge PCR, in which primers are linked to the surface of a solid substrate by a flexible linker. At the end of the PCR reaction are generated different clonal clusters each containing ∼1,000 copies of a single member of the starting library. These clusters are then sequenced. Each sequencing cycle includes the simultaneous addition of a mixture of four fluorescent labeled deoxynucleotides modified with a reversibly terminating moiety at the 3′ position. A modified DNA polymerase drives synchronous extension of primed sequences and then the results are acquired by imaging in four channels.

These strategies allow fast evaluation of absolute miRNA levels and are also able to identify novel miRNAs, but to date they are still costly. In addition, since some errors can be introduced at several steps, limiting the accuracy of the analysis, sequencing results must be validated by alternative methods such as qRT-PCR.

### 3.7. Methods for Detection and Characterization of Circulating miRNAs

**Figure 2.**
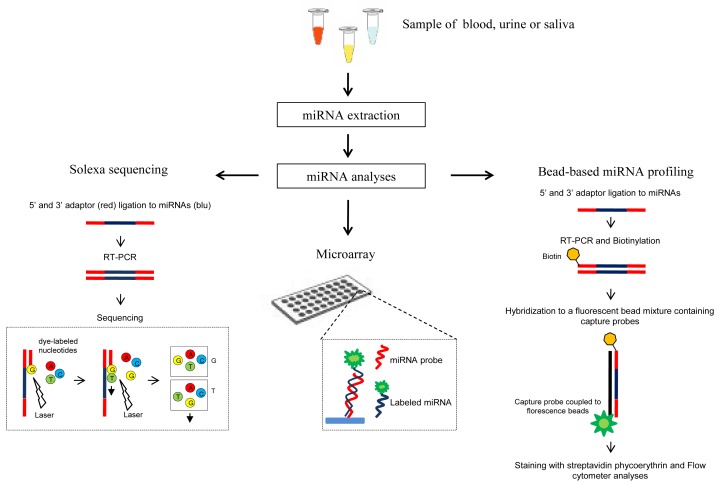
Main methods used for circulating miRNA detection.

Recently, circulating miRNAs are emerging as very promising biomarkers for cancer [29,30], since they are abundant, tissue specific and relatively stable. Thus, several methods have been recently proposed to detect miRNAs in serum, plasma, urine and other human body fluids (Figure 2). Noteworthy the use of body fluids as biological materials for miRNA assays has the main advantage to be noninvasive or minimally invasive approaches.

High-throughput profiling techniques such as Solexa sequencing, miRNA microarray and bead-based miRNA profiling are effective tools for quantification of circulating miRNAs. However, because of the rather high amounts of serum required, high-throughput techniques are mainly used for initial screening analysis. For circulating miRNA detection a higly promising strategy has been recently developed which combines Solexa sequencing or oligonucleotide microarray with the qRT-PCR [33,87,88] thus exploiting the high specificity and sensitivity of the TaqMan stem-loop qRT-PCR based method.

In addition, to make easier the detection of circulating miRNAs, new techniques have been recently developed. By performing miRNA detection through an electrochemical genosensor, Lusi *et al.* [89] were able to directly detect miRNAs without the need of PCR and a labeling reaction, with an assay simple, very fast and ultrasensitive (detection limit of 0.1 pmol). Further developing these and other approaches will certainly enable the application of circulating miRNAs as biomarkers for cancer diagnosis.

However, a common drawback of all these approaches remains the lack of a house-keeping miRNA for normalization of circulating miRNAs that, in contrast to tissue or cellular miRNAs, cannot be normalized against U6 since it is present in a very low concentration in serum and plasma [31,90].

Nevertheless, the normalization of the volume of serum or plasma samples has been proposed as an effective way to overcome such problems. The best approach is to normalize experimental miRNA data using spiked-in synthetic, nonhuman mature miRNA from *C. elegans* or plants as control [91].

## 4. Conclusions

In the last decade the biologic role of miRNAs as “oncomirs” or “tumor suppressors” has generated an enormous expectation for their use in cancer diagnosis, prognosis and treatment. As a consequence the demand for miRNA profiling strategies continues to increase exponentially.

An effective method for miRNA profiling should: (i) involve easy and rapid experimental protocols; (ii) require minimum sample quantity; (iii) have a high specificity and sensitivity with a large measurement dynamic range from sub-femtomolar to nanomolar concentrations and (iv) have low cost. Even if this ideal technique does not yet exist, all the methods summarized in this review address these issues through various strategies.

Furthermore the detection of circulating miRNAs in serum, plasma, urine and other body fluids is now emerging as a promising novel tool to improve cancer screening. Although the field of circulating RNA research is still in its infancy, they show a great potential since they are stable molecules, easily accessible and they can be collected in a relatively noninvasive manner.

## Figures and Tables

**Table 1.** Circulating miRNAs upregulated in cancer.

**miRNA**	**Body Fluids**	**Diseases**	**References**
miR-155, miR-210, miR-21	Serum	Diffuse large B-cell lymphoma	[31]
miR-141	Plasma	Prostate cancer	[32]
miR-25, miR-223	Serum	NSCLC	[33]
miR-155	Serum	Breast cancer	[34]
miR-155, miR-21	Plasma (exosomes)	Lung cancer	[35]
miR-21, miR-141, miR-200 family	Plasma (exosomes)	Ovarian cancer	[36]
miR-17-3p, miR-92	Serum	Colorectal cancer	[37]
miR-126, miR-182	Urine	Bladder cancer	[38]
miR-125a, miR-200a	Saliva	Oral squamous cell carcinoma	[39]

**Table 2.** Comparison of various methods for miRNA profiling

	**Method**	**Sensitivity**	**Specificity**	**Throughput**	**Cost**	**Relevant Features**
**Microarray-based**	**Microarray**	Low	Low	High	Relatively High	Can be used for clinical diagnosis Can only measure miRNA relative abundance Requires 0.2–2 μg of total RNA Specificity and sensitivity can be improved by LNA modification of probes
**Nanotechnology-based**	**ENT**	High	High	Medium	High	Capable of identifying miRNAs with < 2 fold difference in expression level Detection limit at fM level Requires sophisticated instruments No amplification and no labeling are required
	**SPRI**	High	High	Medium	High	Detection limit at attomole level Requires sophisticated instruments No amplification and no labeling are required
	**Gold nanoparticles-based**	High	High	Low	Relatively Low	Relatively simple Detection limit at fM level Does not require sophisticated instruments No amplification and no labeling are required
	**SERS**	High	High	Low	High	Simple to perform Requires sophisticated instruments No amplification and no labeling are required Detection limit at fM level Complicated data interpretation
**qRT-PCR-based**	**Stem-loop qRT-PCR**	High	High	Low	High	Can be used for clinical diagnosis Can be multiplex for high- throughput Specific for mature miRNA Requires only few pg of starting RNA Very sensitive to mismatches
	**SYBR Green qRT-PCR**	High	High	Low	High	Can be used for clinical diagnosis Can be multiplex for high- throughput Specific for mature miRNA Detection limit at fM level
	**miR-Q**	High	High	Low	Low	Can be used for clinical diagnosis Specific for mature miRNA Detection limit at fM level
	**Poly(A)-Tailed Universal RT**	High	Medium	Low	Low	Can be used for clinical diagnosis Requires only few pg of starting RNA
	**Molecular beacons**	Medium	High	Medium	Low	Can be used for clinical diagnosis Simple to perform Detection limit at low nM level Can be used for mature miRNA detection Very sensitive to mismatches
**Amplification-based**	**Padlock-probes and rolling-circle amplification**	Medium	High	Low	Low	Requires radioactive labeling Very sensitive to mismatches Requires few ng of starting RNA
	**Bead-based flow cytometry**	Medium	High	High	Low	Can be used for clinical diagnosis Detection limit at pM level Requires sophisticated instruments
**Enzymatic-based**	**Splinted ligation**	Low	High	Medium	Low	Requires radioactive labeling Requires from ng to μg of starting RNA
	**ELONA**	Medium	Medium	High	Low	Requires very short time Requires more than 30 ng of starting RNA
	**Bioluminescence detection**	High	Medium	High	Low	Simple and rapid Detection limit at fM level
	**RAKE**	High	High	High	Medium	Relatively complex Requires as low as 10 pg of starting RNA
	**Invader assay**	Medium	High	Low	Low	Can be modified for high throughput very simple and rapid able to discriminate between precursor and mature miRNAs Very sensitive to mismatches Requires at least 50 ng of total RNA
**Deep sequencing-based**	**454 pyrosequencing**	Low	High	High	High	Allow also to discovery new miRNAs Requires multiple steps Results must be validated by alternative methods Requires at least 2–10 μg of total RNA
	**SOLiD**	Low	High	High	High	Allow also to discovery new miRNAs Requires multiple steps Results must be validated by alternative methods Requires at least 2–10 μg of total RNA
	**Solexa**	Low	High	High	High	Allow also to discovery new miRNAs Requires multiple steps Results must be validated by alternative methods Requires at least 2–10 μg of total RNA
